# Supplementary material for: What Should I do and Who’s to blame? A cross-national study on youth’s attitudes and beliefs in times of COVID-19
Source: PLoS One. 2022 Dec 21;17(12):e0279366. doi: 10.1371/journal.pone.0279366 (PMC9770422; doi:10.1371/journal.pone.0279366)
Supplement: S1 Table — (DOCX) [file pone.0279366.s001.docx]

What Should I do and Who's to Blame? A Cross-National Study on Youth’s Attitudes and Beliefs in Times of COVID-19

Supplementary Material

Elisabeth L. de Moor, Ting-Yu Cheng, Jenna E. Spitzer, Christian Berger, Alexia Carrizales, Claire F. Garandeau, Maria Gerbino, Skyler T. Hawk, Goda Kaniušonytė, Asiye Kumru, Elisabeth Malonda, Anna Rovella, Yuh-Ling Shen, Laura K. Taylor, Maarten van Zalk, Susan Branje, Gustavo Carlo, Laura Padilla Walker, & Jolien Van der Graaff*

* Corresponding author

Table S1

*Descriptive statistics and demographics per country*

|  | Argentina (*n* = 253) | | Chile (*n* = 146) | | China (*n* = 286) | | Finland (*n* = 465) | | France (*n* = 506) | |
| --- | --- | --- | --- | --- | --- | --- | --- | --- | --- | --- |
|  | *M* (*SD*)/  Frequency (%) | Range | *M* (*SD*)/  Frequency (%) | Range | *M* (*SD*)/  Frequency (%) | Range | *M* (*SD*)/  Frequency (%) | Range | *M* (*SD*)/  Frequency (%) | Range |
| Age | 21.14 (2.35) | 18–25 | 21.55 (2.01) | 18–25 | 21.62 (2.16) | 18–25 | 22.01 (2.20) | 18–25 | 21.69 (2.05) | 18–25 |
| Gender (female) | 184 (73.3%) | – | 117 (81.3%) | – | 147 (51.6%) | – | 377 (84.3%) | – | 422 (85.4%) | – |
| COVID-19 burden | 3.10 (0.72) | 1.22–4.78 | 3.26 (0.63) | 1.67–4.78 | 3.28 (0.66) | 1.44–5 | 2.86 (0.68) | 1–4.56 | 2.96 (0.76) | 1.11–4.89 |
| Individualistic values | 4.48 (0.84) | 1.38–6.63 | 4.28 (0.87) | 2–6.50 | 5.22 (0.74) | 2.13–7 | 4.01 (0.76) | 1.38–6.38 | 4.34 (0.96) | 1.75–6.88 |
| Collectivistic values | 5.49 (0.74) | 3.25–7 | 5.50 (0.63) | 3.50–7 | 5.19 (0.74) | 1.50–6.75 | 5.12 (0.67) | 1.7–6.50 | 5.07 (0.80) | 2.13–7 |
| Empathy | 3.87 (0.50) | 1.40–5 | 3.95 (0.42) | 2.50–4.90 | 3.69 (0.48) | 1.60–5 | 3.96 (0.59) | 1.30–5 | 3.68 (0.49) | 1.70–4.70 |
| Social identification | 3.26 (0.88) | 1–5 | 3.28 (0.88) | 1–5 | 3.88 (0.62) | 1.50–5 | 3.16 (0.88) | 1–5 | 2.73 (0.87) | 1–5 |
| Attitude towards government approach | 3.03 (1.06) | 1–5 | 2.48 (1.09) | 1–5 | – | – | 4.12 (0.66) | 1.67–5 | 2.98 (1.01) | 1–5 |
| Blaming of certain groups | 1.24 (0.39) | 1–3 | 1.15 (0.31) | 1–2.67 | 1.66 (0.61) | 1–4 | 1.21 (0.32) | 1–3 | 1.31 (0.51) | 1–4 |
| Cumulative cases per 100,000 inhabitants* | 1,079.76 | – | 2,459.58 | – | 5.95 | – | 178.53 | – | 788.65 | – |
| Strictness of approach* | 89.53 | – | 83.31 | – | 78.24 | – | 34.31 | – | 49.20 | – |
| National individualistic orientation* | 46.00 | – | 23.00 | – | 20.00 | – | 63.00 | – | 71.00 | – |

Table S1 (continued)

|  | Germany (*n* = 375) | | Ireland (*n* = 495) | | Italy (*n* = 502) | | Lithuania (*n* = 346) | | Spain (*n* = 179) | |
| --- | --- | --- | --- | --- | --- | --- | --- | --- | --- | --- |
|  | *M* (*SD*)/  Frequency (%) | Range | *M* (*SD*)/  Frequency (%) | Range | *M* (*SD*)/  Frequency (%) | Range | *M* (*SD*)/  Frequency (%) | Range | *M* (*SD*)/  Frequency (%) | Range |
| Age | 22.04 (1.95) | 18–25 | 21.57 (2.07) | 18–25 | 21.86 (1.90) | 18–25 | 19.90 (1.80) | 18–25 | 20.56 (1.67) | 18–25 |
| Gender (female) | 295 (79.1%) | – | 380 (80.0%) | – | 320 (64.0%) | – | 253 (74.0%) | – | 150 (84.7%) | – |
| COVID-19 burden | 3.04 (0.70) | 1–5 | 3.35 (0.72) | 1.44–5 | 3.22 (0.76) | 1–5 | 3.22 (0.83) | 1–5 | 3.35 (0.73) | 1–5 |
| Individualistic values | 4.15 (0.78) | 2.13–6.38 | 4.52 (0.78) | 2.38–6.63 | 4.71 (0.82) | 2.38–6.88 | 4.73 (0.77) | 2.50–6.88 | 4.25 (0.84) | 1–6.75 |
| Collectivistic values | 5.02 (0.73) | 2.63–6.75 | 5.26 (0.74) | 2.38–7 | 5.34 (0.73) | 2.25–6.88 | 5.25 (0.88) | 2.50–7 | 5.44 (0.79) | 1–7 |
| Empathy | 3.91 (0.52) | 1.80–5 | 4.06 (0.55) | 2.10–5 | 3.83 (0.56) | 1.80–5 | 3.72 (0.52) | 1.50–5 | 4.07 (0.47) | 1.80–5 |
| Social identification | 2.91 (0.93) | 1–4.75 | 3.32 (0.89) | 1–5 | 3.30 (0.92) | 1–5 | 2.98 (0.83) | 1–5 | 3.66 (0.88) | 1–5 |
| Attitude towards government approach | 3.93 (0.74) | 1–5 | 3.73 (0.97) | 1–5 | 3.62 (0.87) | 1–5 | 3.66 (0.90) | 1–5 | 3.01 (1.06) | 1–5 |
| Blaming of certain groups | 1.15 (0.32) | 1–3 | 1.17 (0.31) | 1–3 | 1.33 (0.45) | 1–3.33 | 1.45 (0.58) | 1–4 | 1.14 (0.31) | 1–3 |
| Cumulative cases per 100,000 inhabitants* | 249.09 | – | 527.87 | – | 408.78 | – | 164.93 | – | 1,747.74 | – |
| Strictness of approach* | 56.79 | – | 44.82 | – | 57.36 | – | 28.18 | – | 59.17 | – |
| National individualistic orientation* | 67.00 | – | 70.00 | – | 76.00 | – | 60.00 | – | 51.00 | – |

Table S1 (continued)

|  | Taiwan (*n* = 169) | | The Netherlands  (*n* = 472) | | Turkey (*n* = 372) | | United States of America (*n* = 1,116) | |
| --- | --- | --- | --- | --- | --- | --- | --- | --- |
|  | *M* (*SD*)/  Frequency (%) | Range | *M* (*SD*)/  Frequency (%) | Range | *M* (*SD*)/  Frequency (%) | Range | *M* (*SD*)/  Frequency (%) | Range |
| Age | 21.38 (1.84) | 18–25 | 21.36 (2.36) | 18–25 | 21.62 (1.92) | 18–25 | 21.61 (2.17) | 18–25 |
| Gender (female) | 113 (66.9%) | – | 270 (57.9%) | – | 253 (68.4%) | – | 533 (49.4%) | – |
| COVID-19 burden | 3.11 (0.86) | 1–5 | 2.86 (0.66) | 1–4.89 | 3.88 (0.72) | 1–5 | 3.11 (0.84) | 1–5 |
| Individualistic values | 4.97 (0.69) | 3.38–7 | 4.39 (0.79) | 1–7 | 5.03 (0.88) | 2.63–6.88 | 4.62 (0.83) | 1–7 |
| Collectivistic values | 5.03 (0.73) | 3.38–6.75 | 4.89 (0.76) | 1–7 | 5.60 (0.81) | 1.63–7 | 5.01 (0.94) | 1.63–7 |
| Empathy | 3.77 (0.45) | 2.50–4.90 | 3.69 (0.54) | 1.80–4.90 | 4.01 (0.57) | 2.10–5 | 3.75 (0.66) | 1.70–5 |
| Social identification | 3.52 (0.58) | 1–5 | 2.98 (0.74) | 1–5 | 3.46 (1.03) | 1–5 | 3.05 (0.95) | 1–5 |
| Attitude towards government approach | 4.06 (0.58) | 2–5 | 3.71 (0.70) | 1–5 | 3.18 (1.08) | 1–5 | 3.02 (1.20) | 1–5 |
| Blaming of certain groups | 1.69 (0.54) | 1–3 | 1.46 (0.65) | 1–4 | 1.72 (0.64) | 1–4 | 1.42 (0.66) | 1–4 |
| Cumulative cases per 100,000 inhabitants* | 2.23 | – | 310.19 | – | 288.24 | – | 992.76 | – |
| Strictness of approach* | 23.15 | – | 46.29 | – | 55.57 | – | 68.98 | – |
| National individualistic orientation* | 17.00 | – | 80.00 | – | 37.00 | – | 91.00 | – |

*Note*. Percentages of females reflect the percentage of all individuals who reported their gender.
* Only one score per country.
