# Supplementary material for: What Should I do and Who’s to blame? A cross-national study on youth’s attitudes and beliefs in times of COVID-19
Source: PLoS One. 2022 Dec 21;17(12):e0279366. doi: 10.1371/journal.pone.0279366 (PMC9770422; doi:10.1371/journal.pone.0279366)
Supplement: S2 Table — (DOCX) [file pone.0279366.s002.docx]

What Should I do and Who's to Blame? A Cross-National Study on Youth’s Attitudes and Beliefs in Times of COVID-19

Supplementary Material

Elisabeth L. de Moor, Ting-Yu Cheng, Jenna E. Spitzer, Christian Berger, Alexia Carrizales, Claire F. Garandeau, Maria Gerbino, Skyler T. Hawk, Goda Kaniušonytė, Asiye Kumru, Elisabeth Malonda, Anna Rovella, Yuh-Ling Shen, Laura K. Taylor, Maarten van Zalk, Susan Branje, Gustavo Carlo, Laura Padilla Walker, & Jolien Van der Graaff*

* Corresponding author

Table S2

*Newly designed perceived COVID-19 burden measure used in the present study*

| Please indicate if you have worried or were burdened by the following situations during the COVID-19 pandemic. | |
| --- | --- |
| 1. | … being or become infected with or having symptoms of COVID-19 |
| 2. | … friends or family being or becoming infected with COVID-19 |
| 3. | … feeling or being restricted to leave your home |
| 4. | … being restricted in visiting friends or family |
| 5. | … not being able to perform leisure activities |
| 6. | … tensions at home or family conflict |
| 7. | … experiencing delays/obstacles in work or study |
| 8. | … being at an increased risk for lower financial means |
| 9. | … (threat of) job loss or insolvency of private company for you or someone in your household |

*Note*. Items were rated on a Likert scale from 1 (*not at all burdensome*) to 5 (*very burdensome*).
