# Supplementary material for: What Should I do and Who’s to blame? A cross-national study on youth’s attitudes and beliefs in times of COVID-19
Source: PLoS One. 2022 Dec 21;17(12):e0279366. doi: 10.1371/journal.pone.0279366 (PMC9770422; doi:10.1371/journal.pone.0279366)
Supplement: S4 Table — (DOCX) [file pone.0279366.s004.docx]

What Should I do and Who's to Blame? A Cross-National Study on Youth’s Attitudes and Beliefs in Times of COVID-19

Supplementary Material

Elisabeth L. de Moor, Ting-Yu Cheng, Jenna E. Spitzer, Christian Berger, Alexia Carrizales, Claire F. Garandeau, Maria Gerbino, Skyler T. Hawk, Goda Kaniušonytė, Asiye Kumru, Elisabeth Malonda, Anna Rovella, Yuh-Ling Shen, Laura K. Taylor, Maarten van Zalk, Susan Branje, Gustavo Carlo, Laura Padilla Walker, & Jolien Van der Graaff*

* Corresponding author

Table S4

*Constrained multigroup mediation model with attitude towards government approach and blaming of certain groups regressed on COVID-19 burden, individualistic values, collectivistic values, and empathy, mediated by social identification; grouping on strictness of the national COVID-19 approach*

|  | ***Direct effects*** | | | | | ***Indirect effects*** | | | | |
| --- | --- | --- | --- | --- | --- | --- | --- | --- | --- | --- |
|  | *b* | SE(*β*) | *β* | | *p* | *b* | SE(*β*) | | *β* | *p* |
| **Predictors of attitude towards government approach** | | | | | |  | |  |  |  |
| COVID-19 burden | -0.13 | .02 | -0.12 | < .001 | | 0.02 | < .01 | | 0.02 | < .001 |
| Individualistic values | 0.01 | .02 | 0.01 | .442 | | < -0.01 | < .01 | | < -0.01 | .273 |
| Collectivistic values | 0.09 | .02 | 0.09 | < .001 | | 0.06 | .01 | | 0.06 | < .001 |
| Empathy | 0.05 | .02 | 0.03 | .070 | | 0.05 | .01 | | 0.03 | < .001 |
| Social identification | 0.19 | .02 | 0.20 | < .001 | | – | – | | – | – |
| **Predictors of blaming of certain groups** | | |  |  | |  |  | |  |  |
| COVID-19 burden | 0.10 | .01 | 0.15 | < .001 | | < 0.01 | < .01 | | 0.01 | < .001 |
| Individualistic values | 0.11 | .01 | 0.17 | < .001 | | < 0.01 | < .01 | | < -0.01 | .287 |
| Collectivistic values | 0.01 | .01 | 0.01 | .641 | | 0.01 | < .01 | | 0.02 | < .001 |
| Empathy | -0.21 | .02 | -0.24 | < .001 | | 0.01 | < .01 | | 0.01 | < .001 |
| Social identification | 0.03 | .01 | 0.06 | < .001 | | – | – | | – | – |
| **Predictors of social identification** | | |  |  | |  |  | |  |  |
| COVID-19 burden | 0.12 | .01 | 0.11 | < .001 | | – | – | | – | – |
| Individualistic values | -0.01 | .01 | -0.01 | .271 | | – | – | | – | – |
| Collectivistic values | 0.31 | .01 | 0.28 | < .001 | | – | – | | – | – |
| Empathy | 0.25 | .01 | 0.17 | < .001 | | – | – | | – | – |
| **Covariances** |  |  |  |  | |  |  | |  |  |
| Attitude towards government approach × Blaming of certain groups | < -0.01 | .02 | -0.01 | .721 | | – | – | | – | – |

*Note*. *R*^2^ for attitude towards government approach = 6.2%, *R*^2^ for blaming of certain groups = 17.6%, *R*^2^ for social identification = 10.6%
