# Supplementary material for: What Should I do and Who’s to blame? A cross-national study on youth’s attitudes and beliefs in times of COVID-19
Source: PLoS One. 2022 Dec 21;17(12):e0279366. doi: 10.1371/journal.pone.0279366 (PMC9770422; doi:10.1371/journal.pone.0279366)
Supplement: Attachment — Submitted filename: Response to Reviewers_COVID-19_R1.docx [file pone.0279366.s006.docx]

**Editor**

1. **Please explain how the scales were translated into the language of the participants’ country? It is also unclear whether the measures of culture orientation, empathy and social identification have been validated for use in the 14 study countries.**

Reply: Where possible, we used measures that had already been translated and used in cross-national research, such as the Culture Orientation Scale (COS; Triandis & Gelfland, 1998) to measure collectivistic and individualistic values, and the Interpersonal Reactivity Index (IRI; Davis, 1983). The IRI was available for use in all 14 countries and the COS was available for use in 10 out of 14 countries in our sample. The measures on attitude towards government approach, blaming of certain groups for COVID-19, social identification, and perceived COVID-19 burden where either adapted or designed for this project. The collaborators of each of the countries translated these questionnaires to the native language. We now briefly explain this procedure in the Method section:

“Participants completed an online survey throughout the summer and fall of 2020 in the primary language of their county. For several of the questionnaires included, versions in the primary languages were already available; questionnaires for which no primary language version existed were translated by collaborators from each of these countries.” (p. 8)

2. **Although individualism/collectivism is perhaps the most commonly applied construct in explaining cultural effects, other dimensions of culture identified by Hofstede, e.g. power distance, masculinity-femininity uncertainty avoidance, long- vs. short-term orientation and indulgence-restraint, may also be relevant. It would be useful if the authors acknowledge the need for further cross-cultural research on this topic.**

Reply: We thank you for this suggestion. Of course, we agree that the absence of country-level differences found in the present study based on the cumulative number of COVID-19 cases, the strictness of the national approach, and the national individualistic orientation does not exclude the possibility of differences based on other country-level factors. We have added nuance to our discussion of country-level moderation and elaborated with some suggestions for future research:

“Alternatively, although there were no country-level differences depending on the cumulative number of COVID-19 cases, the strictness of the national approach, or the national level of individualistic orientation, it is possible that other factors at the country-level may indicate differences in youth’s attitudes towards their government’s approach to combatting the spread of the disease and to their blaming of individuals from certain risk groups, ethnic groups, and countries or regions. For instance, the present study focused on individual and country-level differences in collectivistic and individualistic values, but other cultural dimensions may also play a role (e.g., [20, 56]). As a case in point, differences in the uncertainty avoidance dimension (i.e., discomfort with and resistance to unfamiliar phenomena; [56]) have been associated at the country level with spread of the disease and with greater mortality risk [65, 66]. In addition to health outcomes, higher uncertainty avoidance has been related at the country-level to less gathering of people in public places [67] and more consumer stockpiling [68], and at an individual level to greater beliefs in COVID-19 conspiracy theories [69]. Thus, it may be interesting to examine individual- and country-level values also in relation to youth’s attitudes towards their government’s approach to combatting the spread of the disease and to their blaming of risk groups and individuals from certain ethnic groups and countries or regions.” (p. 21)

**Journal requirements**

1. **Please ensure that your manuscript meets PLOS ONE's style requirements, including those for file naming. The PLOS ONE style templates can be found at**

[**https://journals.plos.org/plosone/s/file?id=wjVg/PLOSOne_formatting_sample_main_body.pdf**](https://journals.plos.org/plosone/s/file?id=wjVg/PLOSOne_formatting_sample_main_body.pdf)  **and**

[**https://journals.plos.org/plosone/s/file?id=ba62/PLOSOne_formatting_sample_title_authors_affiliations.pdf**](https://journals.plos.org/plosone/s/file?id=ba62/PLOSOne_formatting_sample_title_authors_affiliations.pdf)

Reply: We have adjusted the manuscript to the journal required format. These adjustments were made without tracking changes, to keep content adjustments based on the editor and reviewers’ comments clearly visible.

2. **Peer review at PLOS ONE is not double-blinded (**[**https://journals.plos.org/plosone/s/editorial-and-peer-review-process**](https://journals.plos.org/plosone/s/editorial-and-peer-review-process)**). For this reason, authors should include in the revised manuscript all the information removed for blind review.**

Reply: All information has been unblinded.

3. **Thank you for stating the following in the Acknowledgments Section of your manuscript:**

**“Elisabeth de Moor and Susan Branje were supported by a grant from the European Research Council (ERC-CoG INTRANSITION-773023). Jolien Van der Graaff received a grant from the COVID-19 fund of the Faculty of Social Sciences of Utrecht University.”**

**We note that you have provided additional information within the Acknowledgements Section that is not currently declared in your Funding Statement. Please note that funding information should not appear in the Acknowledgments section or other areas of your manuscript. We will only publish funding information present in the Funding Statement section of the online submission form.**

**Please remove any funding-related text from the manuscript and let us know how you would like to update your Funding Statement. Currently, your Funding Statement reads as follows:**

**“JvdG received a grant from the Faculty of Social Sciences of Utrecht University to fund data collection (no grant number available). URL:** [**https://www.uu.nl/en/organisation/faculty-of-social-and-behavioural-sciences**](https://www.uu.nl/en/organisation/faculty-of-social-and-behavioural-sciences)

**ELdM and SB were supported by a grant from the European Research Council (ERC-CoG INTRANSITION-773023). URL:** [**https://erc.europa.eu/**](https://erc.europa.eu/)

**The funders had no role in study design, data collection and analysis, decision to publish, or preparation of the manuscript.”**

**Please include your amended statements within your cover letter; we will change the online submission form on your behalf.**

Reply: We removed the funding information from the Acknowledgements and carefully checked the funding statement. No changes are needed in the funding statement.

4. **In your Data Availability statement, you have not specified where the minimal data set underlying the results described in your manuscript can be found. PLOS defines a study's minimal data set as the underlying data used to reach the conclusions drawn in the manuscript and any additional data required to replicate the reported study findings in their entirety. All PLOS journals require that the minimal data set be made fully available. For more information about our data policy, please see** [**http://journals.plos.org/plosone/s/data-availability**](http://journals.plos.org/plosone/s/data-availability)**.**

**Upon re-submitting your revised manuscript, please upload your study’s minimal underlying data set as either Supporting Information files or to a stable, public repository and include the relevant URLs, DOIs, or accession numbers within your revised cover letter. For a list of acceptable repositories, please see** [**http://journals.plos.org/plosone/s/data-availability#loc-recommended-repositories**](http://journals.plos.org/plosone/s/data-availability#loc-recommended-repositories)**. Any potentially identifying patient information must be fully anonymized.**

**Important: If there are ethical or legal restrictions to sharing your data publicly, please explain these restrictions in detail. Please see our guidelines for more information on what we consider unacceptable restrictions to publicly sharing data:** [**http://journals.plos.org/plosone/s/data-availability#loc-unacceptable-data-access-restrictions**](http://journals.plos.org/plosone/s/data-availability#loc-unacceptable-data-access-restrictions)**. Note that it is not acceptable for the authors to be the sole named individuals responsible for ensuring data access.**

**We will update your Data Availability statement to reflect the information you provide in your cover letter.**

Reply: An anonymized dataset is now made available on the project’s OSF page (<https://osf.io/738z5/>). Both on the OSF page as in the Data Availability statement, we stress that researchers interested in using the data are requested to contact the core research team of the project, given the large amount of people involved in the project and the possibility for overlap in projects.

5. **We note that you have referenced (Unpublished doctoral dissertation, University of Utah and Unpublished manuscript ) which has currently not yet been accepted for publication. Please remove this from your References and amend this to state in the body of your manuscript: (ie “Bewick et al. [Unpublished]”) as detailed online in our guide for authors**

[**http://journals.plos.org/plosone/s/submission-guidelines#loc-reference-style**](http://journals.plos.org/plosone/s/submission-guidelines#loc-reference-style)

Reply: This has been adjusted in the manuscript.

6. **Please include captions for your Supporting Information files at the end of your manuscript, and update any in-text citations to match accordingly. Please see our Supporting Information guidelines for more information:** [**http://journals.plos.org/plosone/s/supporting-information**](http://journals.plos.org/plosone/s/supporting-information)**.**

Reply: Captions for all Supporting Information files have been included on page 31 of the manuscript.

**Reviewer #1**

**the work is interesting and well written. Some aspects that can improve the work are emphasized.**

1. **An in-depth study of country-specific literature in the construction of the theoretical part with respect to the topics under investigation, including the part on the restrictions of different governments.**

Reply: We agree that a greater focus on country-level differences and country-level moderation of the researched associations would strengthen our Introduction. At the same time, we believe an in-depth study of the topics under investigation for all 14 examined countries that would fully delineate the country-level differences in these processes is beyond the scope of the present manuscript. In the first part of our Introduction, we have elaborated on country-level differences:

“Finally, even though previous research has shown links between person characteristics and youth’s prosocial attitudes to be consistent across cultures (e.g. [12]), it is important to take differences in country-level contextual factors into account. For instance, the number of COVID-19 cases varied greatly across countries at the time of this study (e.g., very low in Taiwan and high in Chile), and the approach that governments took in response to the pandemic also varied. As a result, citizens experienced many more restrictions in one country (e.g., Argentina) than they did in another (e.g., Finland). Therefore, we explored differences across countries in the strength of predictors based on cumulative COVID-19 cases, individualistic orientation of the country, and the strictness of the national COVID-19 approach at the time of assessment.” (pp. 3-4)

In addition, we have extended our rationale for examining cumulative COVID-19 cases per 100,000 inhabitants, the strictness of the national approach, and the national-level individualistic orientation as country-level moderators of the researched processes:

“We examined social identification as a mediator and explored moderation at the country level to uncover potential cross-country differences in these associations. Specifically, we examined moderation by the cumulative COVID-19 cases per 100,000 inhabitants, the strictness of the national approach as factors of COVID-19-related country differences, and the national-level individualistic orientation as a more general country difference factor, as the association between individual-level factors may depend on both the specific COVID-19 situation and general values in a country (see Fig 1 for a complete conceptual overview of the tested associations).” (p. 7)

2. **It would be important to test the power of the overall sample and individual countries.**

Reply: We have clarified that the reported power analysis referred to the overall sample, and have added a test for the multigroup analyses. Because the data were not analyzed per country, such a power analysis was not conducted. The adjusted text reads:

“Prior to registering our study, a power analysis was conducted to estimate the required sample size to detect effects in the mediation model and the extended moderated mediation model. With our complete sample of 5,682 participants, a power of .80, and significance at α = .05, effects of f2 = .002 (very small effect size) could be detected for the mediation model. In the country-level multigroup models which had subgroup sizes of at least 1,116 participants (for the “high” group of national-level individualistic orientation), effects of at least f2 = .011 (small effect size) could be detected.” (pp. 10-11)

3. **In addition, the application implications of the work should be developed**

Reply: In line with your suggestion, we have elaborated on the implications of our findings regarding perceived burden (p. 18), collectivistic and individualistic values (pp. 19-20), and empathy (p. 20). For example, with regard to the perceived burden, the conclusion now reads:

“Thus, perceived burden may play both a positive and negative role in youth’s attitudes, depending on whether it is paired with a greater sense of identification with the people around them. It is also possible that the way burden is related to one’s attitudes depends on the type of burden. For instance, experiencing a burden to one’s health (e.g., getting ill) may be tied to greater social identification, whereas experiencing a social burden (e.g., not being able to visit friends) may be tied to less social identification. In the future, it would be interesting to examine the differential effects of these different types of burden on attitudes and behavior. For policy makers, it may be important to keep in mind the negative effect experiencing a burden may have on youth’s attitudes towards the government and other members of society, and on their behavior during a pandemic more generally.” (p. 18)

**Reviewer #2**

**This article discusses a relevant topic in the current context: what are the determinants (or some of them) of young people's attitudes to public health measures put in place during the pandemic. The topic is of global interest and appropriate for a multidisciplinary journal such as PLOS ONE.**

**ABSTRACT:**

1. **The authors identify their research object rather clearly in the abstract, although I suggest describing the results of their model in greater detail and precision. Also, '(...) blaming certain groups' seems too vague phrasing to me. The authors need to be more specific about which "groups" they refer to in the abstract and the text in general (and in the model). Ideally, it would also be helpful to discuss (along the manuscript) what is the meaning of blaming these groups in the youth context of these countries. I also recommend adding a sentence to put the theory of "social identification" in context, so that it is made more understandable in an interdisciplinary context.**

Reply: We thank you for pointing out these unclarities in the abstract; we have adjusted the summary of our findings to clarify the direction of effects and to specify the groups to which blaming pertained. We have also elaborated on the concept of “social identification” to clarify its meaning. The adjusted text now reads:

“The COVID-19 crisis has had a major impact on youth. This study examined factors associated with youth’s attitudes towards their government’s response to the pandemic and their blaming of individuals from certain risk groups, ethnic backgrounds, and countries or regions. In a sample of 5,682 young adults (Mage = 22) from 14 countries, lower perceived burden due to COVID-19, more collectivistic and less individualistic values, and more empathy were associated with more positive attitudes towards the government and less blaming of others. Youth’s social identification with others in the pandemic mediated these associations in the same direction, apart from the COVID-19 burden on attitudes, which had a positive indirect effect. No evidence of country-level moderation was found.” (p. 2)

2. **Keywords should be words, not sentences.**

Reply: We have shortened the keywords. The keywords now read:

“*Keywords*: Young adults; COVID-19 pandemic; Attitudes towards government; Blaming” (p 2)

**INTRODUCTION:**

3. **It seems to me that the authors in this section offer an adequate amount of information, but that it should be better organized. First, the authors seem to arrive at Figure 1 too quickly. This conceptual model should be better justified (the identification of these factors and the mediating mechanisms) and it would also help to make the hypotheses derived or underlying them more explicit. Some hypotheses are made but not formally highlighted.**

**I would thus reorganize the section so that the reader is first explained the rationale for the identification of the determinants and hypotheses. After that, I would offer the conceptual model (which could possibly also be moved to the methodological part as an analysis model).**

Reply: We thank you for pointing out that the placement of Figure 1 was not optimal. We have moved the figure to the “Current Study” section, where all proposed paths are also again explicitly stated. In the section where the model was originally introduced, we now elaborate on the paths underlying the model:

“In the present study, we examined among young adults from 14 countries 1) their attitudes towards their government’s approach to handling the pandemic and 2) the extent to which they blamed individuals from certain risk groups, ethnic backgrounds, and countries or regions for their negative experiences during the pandemic, which reflect their attitudes towards the institutions and individuals in a society and may also predict their behavior. COVID-19-related and more general individual factors were examined to explain individual differences in youth’s attitudes. In particular, we studied whether youth’s perceived burden from the pandemic, their collectivistic and individualistic values, and their empathic tendencies predicted their attitudes. We further investigated whether youth’s social identification with others during the pandemic could (partly) explain these associations. Finally, even though previous research has shown links between person characteristics and youth’s prosocial attitudes to be consistent across cultures (e.g. [12]), it is important to take differences in country-level context factors into account. For instance, the number of COVID-19 cases varied greatly across countries at the time of this study (e.g., very low in Taiwan and high in Chile), and the approach that governments took in response to the pandemic also varied. As a result, citizens experienced many more restrictions in one country (e.g., Argentina) than they did in another (e.g., Finland). Therefore, we explored differences across countries in the strength of predictors based on cumulative COVID-19 cases, individualistic orientation of the country, and the strictness of the national COVID-19 approach at the time of assessment.” (pp. 3-4)

In addition, we have carefully checked the Introduction so that all proposed paths are made explicit.

4. **One recent reference that could help to add in this section on young people:
   Vacchiano, M., (2022). How the First COVID-19 Lockdown Worsened Younger Generations' Mental Health: Insights from Network Theory. Sociological Research Online,** [**https://doi.org/10.1177/13607804221084723**](https://doi.org/10.1177/13607804221084723)

Reply: We have added the suggested publication to the Introduction section:

“Although their physical health risks from the virus were relatively low, young adults experienced disproportionally high social and financial costs due to the restrictions imposed by governments to curb the spread of the virus [1], as well as costs for their mental health [2].” (p. 3)

**METHODS:**

**The methodological part is sufficiently detailed. One fundamental doubt:**

5. **I am not sure it can be said 100% that a level 2 of 14 units does not need a multilevel analysis. I know there is debate on the issue, but as far as I know the best way to measure effects at the country level is through multilevel analysis. According to Austin (2010) even models with 10 level-2 clusters could produce reliable statistical inference (see also Bryan and Jenkins 2016 on this controversial issue).

   I have no expertise to evaluate the strategy that the authors have set up to test the effect of countries. I am not saying it is wrong, I simply don't know it and fully understand it. Personally, I think a multilevel analysis would be sounder.**

Reply: We agree that the debate about multilevel moderation is very complex, and that the answer to the question of when moderation is appropriate depends on a great many factors, amongst which one’s sample size, number of clusters, cluster size, and estimation method (see Hox et al., 2014). To navigate this issue and select the most appropriate analysis for our study, we consulted faculty from the methodology department of Utrecht University about our approach before pre-registering the study. We have checked again with them for the revised manuscript, and have elaborated on our choice to use a multigroup rather than a multi-level approach:

“Next, to examine moderation by country-level variables, we divided the sample into groups of low, medium, and high on each of the country-level factors. As preregistered, this was done because the number of countries was too small to test a multilevel mediation model, for which a minimum number of 50 clusters is suggested when used in a Structural Equation Framework with Maximum Likelihood (ML) estimation [54-55].” (p. 11)

**RESULTS, DISCUSSION, LIMITATIONS:**

**On the rest of the manuscript, I have little to add. It seems to me that the paper brings out some interesting results and that the authors identify well the limitations of their work and the data.**

6. **In general, the paper is publishable with appropriate modifications. First, (1) reorganizing the introductory section and making explicit in a formal way the hypotheses (and so justifying the conceptual model). Second, the (2) authors need to better justify why multilevel analysis is not performed (here a paper** [**https://doi.org/10.1093/esr/jcv059**](https://doi.org/10.1093/esr/jcv059) **that may help). Multilevel analysis could still be necessary in this paper. Also, in the discussion the differences between countries are not mentioned at all, which casts more doubt on the adequacy of the strategy pursued by the authors to capture country effects. I am sure the authors can offer more interesting arguments on this country differences.**

Reply: We thank you for your assessment of our manuscript. We hope we have satisfactorily addressed your first point in our response to your comment #3 and to your second point in response to your comment #5. We have extended our discussion of country-level differences in response to comment #2 of the Editor. Please see the adjusted text there.
